# Efficacy of traditional Chinese herbal fumigation steam therapy combined with functional exercise on post-stroke hemiplegic shoulder pain

**DOI:** 10.12669/pjms.41.11.12907

**Published:** 2025-11

**Authors:** Guoying Qiu, Liqin He

**Affiliations:** 1 Guoying Qiu Department of Critical Care Medicine, Suzhou Hospital of Integrated Traditional Chinese and Western Medicine, Suzhou, Jiangsu Province 215101, P.R. China; 2 Liqin He Department of Pain Rehabilitation, Suzhou Hospital of Integrated Traditional Chinese and Western Medicine, Suzhou, Jiangsu Province 215101, P.R. China

**Keywords:** Chinese herbal fumigation steam therapy, Functional exercise, Post-stroke, Hemiplegic shoulder pain

## Abstract

**Objective:**

To evaluate the effect of Chinese herbal fumigation steam therapy (CHFST) combined with functional exercise on post-stroke hemiplegic shoulder pain (HSP).

**Methodology:**

This is a retrospective case-control analysis, with case data selected from 136 HSP patients who received treatment at Suzhou Hospital of Integrated Traditional Chinese and Western Medicine from January 2024 to January 2025. Among them, 68 patients who received Chinese herbal fumigation steam therapy (CHFST) combined with functional exercise were matched in a 1:1 ratio with a cohort receiving functional exercise alone, both in addition to routine care. The changes in shoulder pain, shoulder joint range of motion and upper limb motor function, as well as the World Health Organization Quality of Life-BREF (WHOQOL-BREF) scores of the two groups, were evaluated after four weeks of intervention.

**Results:**

The degree of shoulder joint pain in the CHFST group was significantly lower than that in the control group after two and four weeks (P<0.05). The range of motion for shoulder flexion, extension, abduction and external rotation in the CHFST group was higher and the FMA-UE score of upper limb motor function was better than that in the control group after four weeks (P<0.05). Similarly, after four weeks, the WHOQOL-BREF score in the CHFST group was significantly higher compared to the control group (P<0.05).

**Conclusions:**

The combination of CHFST and functional exercise therapy for HSP can more effectively alleviate pain, improve upper limb motor function, shoulder joint mobility and quality of life of patients than the routine treatment regimen.

## INTRODUCTION

Stroke is a significant challenge facing modern medicine, as it can often lead to physical and mental disability and is associated with a heavy socioeconomic burden.^1^,^2^ Stroke is one of the prevalent causes of hemiplegia,^3^ and of hemiplegic shoulder pain (HSP) that occurs in 47% of stroke survivors 1-3 months after the event and is considered one of the most critical risk factors for stroke-associated disability.^1^,^2^,^4^ HSP has a profound negative impact on the rehabilitation plans, psychological well-being and quality of life (QOL) of patients.^2^,^4^,^5^ At present, the clinical management of HSP mainly includes measures such as taping, sub-compartmental steroid injection, suprascapular nerve block, botulinum toxin injection, electrical stimulation, pulse radio frequency therapy and acupuncture and moxibustion.^6^-^13^

However, these measures are associated with a variety of adverse effects. For instance, taping, which includes applying a thin elastic cotton tape to the skin to reduce mechanical retention force and alleviate tensile pain, can easily lead to disuse atrophy of muscles.^7^ Injecting glucocorticoids (such as lidocaine and dexamethasone) into the subacromial space is only effective for 2-4 weeks, while the long-term use increases the risk of tendon degeneration and rupture.^8^ A suprascapular nerve block, which involves injecting local anesthetics around the suprascapular nerve to block pain signal transmission and quickly relieve shoulder pain, can lead to bleeding, local numbness, or weakness in the rotator cuff muscles.^9^ Botulinum toxin injection, which inhibits acetylcholine release and reduces spasticity, requires regular injections with a high treatment cost.^10^

Electrical stimulation, including neuromuscular electrical stimulation (NMES), transcutaneous electrical nerve stimulation (TENS) and functional electrical stimulation (FES), stimulates nerves or muscles through electrical currents to improve local blood circulation and relieve pain.^11^ However, in some patients, long-term treatment may lead to the development of neuromuscular tolerance, resulting in a gradual weakening of the effect.^11^,^12^ Pulse radiofrequency, a non-destructive neuromodulation method that can apply high-voltage pulsed energy near nerves, thereby reducing patients’ pain perception, requires long-term treatment, is expensive and does not address the root cause of the condition.^11^,^12^ While acupuncture and moxibustion can stimulate the meridian qi, promote the movement of qi and blood, relieve the blockage of the meridians and relieve pain by stimulating the shoulder and related meridian points (such as Jianliao, Jianliao, Tianzong, Quchi, etc.), it is associated with adverse events such as dizziness, infection and bleeding.^13^

While several systematic reviews have analyzed the effects of different intervention measures in the treatment of HSP, due to the complex etiology, the clinical importance of these techniques remains uncertain.^6^,^14^,^15^ Moreover, the impact of different rehabilitation interventions on HSP is still controversial.^16^ Chinese herbal fumigation steam therapy (CHFST) is a method that promotes blood circulation, unblocks meridians, reduces swelling and relieves pain through the thermal stimulation and medicinal penetration of local tissues using drug vapor or liquid medicine.^15^,^16^ It is an essential component of traditional Chinese medicine (TCM) external treatment methods. At present, the effectiveness and safety of CHFST have been confirmed in dermatology, ophthalmology and orthopedics.^16^,^17^

In addition, the CHFST formula applied in this study is not a single classical prescription but a modified empirical formula derived from the traditional principles of activating blood circulation, dispelling wind, and relieving pain as described in ancient texts such as Bei Ji Qian Jin Yao Fang and Shang Han Lun. Over the past decade, it has been refined and standardized in our hospital’s rehabilitation and pain departments, where it has been widely used in patients with musculoskeletal pain and post-stroke complications. This retrospective case-control analysis aimed to assess the efficiency of CHFST in the treatment of HSP.

## METHODOLOGY

The clinical data of HSP patients who received treatment at Suzhou Hospital of Integrated Traditional Chinese and Western Medicine from January 2024 to January 2025 were retrospectively analyzed.

### Ethical Approval:

The ethics committee of our hospital approved this study with the number: 2025-024; Date: July 11^th^ 2025.

### Inclusion criteria:

- Meets the diagnostic criteria for stroke per 2021 Chinese Guidelines for the Diagnosis and Treatment of Acute Ischemic Stroke. Stroke was confirmed by cranial CT/MRI.
- First onset of HSP.^4^
- At least one subjective report of hemiplegic shoulder joint pain with a Visual Analog Scale (VAS) score ≥ 3.
- Age ≥ 18 years.
- Complete clinical data.

### Exclusion criteria:

- Traumatic diseases such as shoulder joint fractures and dislocations.
- Previous rotator cuff disease, frozen shoulder, thoracic outlet syndrome, osteoarthritis and bursitis.
- Severe liver and kidney dysfunction, coagulation dysfunction.
- Patients with cognitive impairment.

### Treatments:

### Conventional treatment:

measures to reduce intracranial pressure, improve cerebral blood circulation, promote brain cell metabolism, nourish brain nerves and control intracranial edema. Special attention was paid to regulating the patient’s blood lipids, blood pressure, blood sugar, etc.

### Functional exercise:

### Posture therapy:

- Lying on the uninjured side, the patients were instructed to fully extend the shoulder joint and bend it as much as possible by 90 degrees. The joints of the elbow, wrist and fingers were then stretched separately.
- When lying on the affected side, the patients fully extended the shoulder joint forward. The elbow joint, wrist dorsum extension joint and finger joint were then extended separately.
- When lying in the supine position and able to tolerate pain or swelling, patients were instructed to extend their scapulae as far forward as possible. The upper limbs, including the elbow joints, wrists and fingers, were then stretched separately. A pad was placed under the scapula on the affected side to aid the patient’s recovery.
- When in sitting position, the patients were guided to place the affected limb on the chest frame, keeping the scapula extended forward to prevent limb prolapse.
- ***Passive movement:*** The patient repeated passive movement of the shoulder, elbow, wrist and finger joints of the affected limb 10 times in each direction, twice a day, with no obvious pain. During the joint movement, attention was paid to venous return. The affected limb was raised appropriately to avoid congestion.
- ***Active exercise:*** The healthy limb was used to drive the affected side to perform upward and outward exercises, three times a day for 15 minutes each time. These functional exercise protocols-including frequency, duration, and therapist supervision-were identical in both groups. In the CHFST group, functional exercise was conducted within 30 minutes following each steam fumigation session to enhance analgesic and relaxation effects. The duration of treatment was one month.

### TCM Formula:

15g Chuanxiong Rhizome (Ligustici Chuanxiong Rhizoma), 15g Safflower (Carthami Flos), 20g Duhuo (Angelicae Pubescentis Radix), 15g Qianghuo (Notopterygii Rhizoma et Radix), 30g Shenjincao (Field Horsetail Herb), 30g Chuangucao (Speranskia Tuberculata Herb), 20g frankincense (Olibanum), 15g myrrh (Commiphora myrrha Eng1.), 15g Cinnamon Twig (Cinnamomi Ramulus), 20g Asarum (Asari Radix et Rhizoma), 15g Mugwort Leaf (Artemisiae Argyi Folium), 15g Mulberry Twig (Mori Ramulus), 15g Angelica (Angelicae Sinensis Radix), 15g Weilingxian (Clematidis Radix et Rhizoma), 15g Papaya Fruit (Chaenomelis Fructus), 100g ginger (Zingiber officinale) , 100g Rice Wine and 100g salt.

### CHFST

To prepare the solution, 1500 ml of medication was added to water, soaked for 30 minutes, boiled, simmered over low heat for 20 minutes and filtered. The extract was collected and poured into the fumigation basin; the temperature was maintained at 40-45°C (suitable for patient tolerance). First, the shoulder was steam fumigated with medication for 10 minutes. A towel was then soaked in the medicine and applied to the affected area, 20 minutes each time, twice in the morning and twice in the evening. Treatment lasted for a total of four weeks.

### Collected data:

All ROM measurements were performed using a standard goniometer by rehabilitation physicians trained in joint mobility assessment. VAS scoring was also conducted by the same team using standard clinical protocols. While all assessors received structured training, no formal inter- or intra-rater reliability analysis (e.g., ICCs) was conducted due to the retrospective nature of the study. Moreover, the assessors were not blinded to treatment allocation, which may have introduced expectation bias. The following information was collected from all patients:

- Basic clinical data, including gender, age, body mass index (BMI), complications (hypertension, diabetes, coronary heart disease), smoking status, alcohol consumption, stroke type (cerebral infarction, cerebral hemorrhage), affected side and stroke course.
- The degree of pain. At baseline, after two and four weeks of intervention, the VAS scoring system was used to assess the severity of shoulder pain in patients, with scores ranging from 0 to 10 (the higher the score, the more severe the pain).
- Shoulder joint mobility, upper limb function and QOL. The range of motion (ROM) of the shoulder joint was measured using a joint angle ruler. Joint range of motion in the directions of shoulder flexion, extension, abduction and external rotation that are closely related to post-stroke HSP were selected for evaluation. The reference values were: shoulder flexion (0-170°), shoulder extension (0-60°), shoulder abduction (0-170°) and shoulder external rotation (0-90°). Higher degrees reflected higher joint mobility. The upper limb function was evaluated using the Fugl Meyer Upper Limb Function Score (FMA-UE, 0-66 points, with higher scores indicating better function).
- ***QOL:*** The World Health Organization Quality of Life-BREF (WHOQOL-BREF) was used to assess the QOL of patients. The assessment included four fields: physical health, psychological, social and environmental domains, with a total of 24 questions in these four fields. In addition, two subjective feelings about QOL and health status were assessed, totaling 26 questions. Each question was rated from 1 to 5 points, with a maximum score of 130 points. A higher score indicated better QOL.

### Statistical Analysis:

The SPSS/PC statistical software (version 25.0; IBM Corp, Armonk, NY, USA) for Windows was used. The count data were presented in the form of n (%) and the differences between groups were analyzed using the chi-square test. Visual (histogram and probability plot) and analytical (Kolmogorov-Smirnov/Shapiro-Wilk test) methods were used to evaluate whether variables follow a normal distribution. The measurement data that conforms to normal distribution were expressed in the form of mean ± standard deviation (SD) and an independent sample t-test was used to compare the differences between the two groups. Age and BMI were summarized using descriptive statistics and compared between groups using independent sample t-tests. WHOQOL-BREF scores were assessed using paired t-tests for within-group comparisons, and Mann-Whitney U tests for between-group comparisons. Non normally distributed data were represented by median and interquartile range. The Mann-Whitney U test was used for intergroup comparison and the Wilcoxon signed rank test was used for intra-group comparison of stroke duration, shoulder joint range of motion and upper limb function score. For VAS scores collected at three time points (baseline, week two, week four), due to minor missing data and limited sample size, we did not apply mixed-effects modeling. Instead, non-parametric tests were used to assess within- and between-group trends. Similarly, for ROM and FMA-UE outcomes at week 4, ANCOVA was not applied due to retrospective design and baseline variation; comparisons were made using Mann-Whitney U tests. The statistical significance was P<0.05. PRISM 8.0 software (GraphPad, San Diego, USA) was used to prepare charts.

## RESULTS

**Fig.1:**
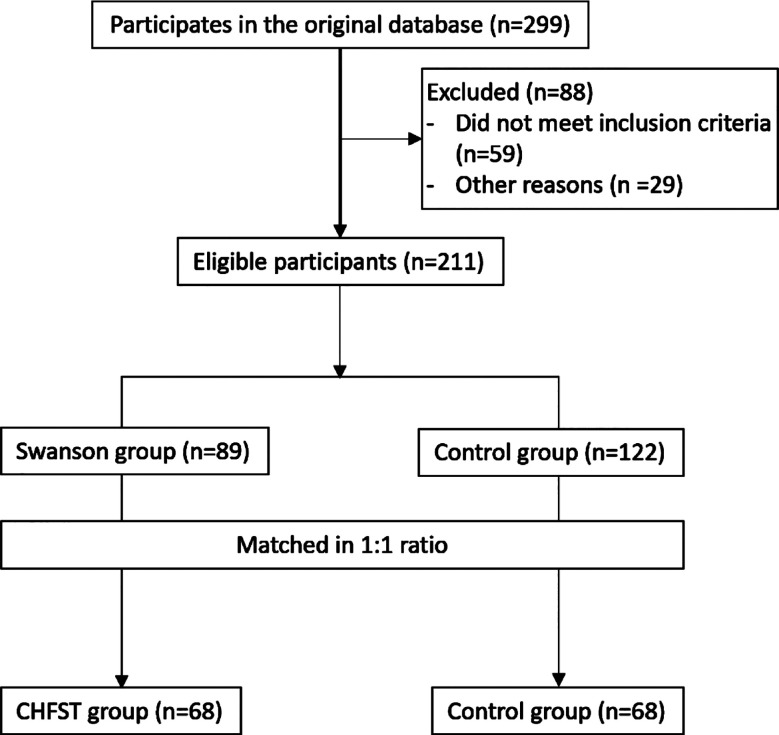
Patient Screening Process Diagram.

**Fig.2:**
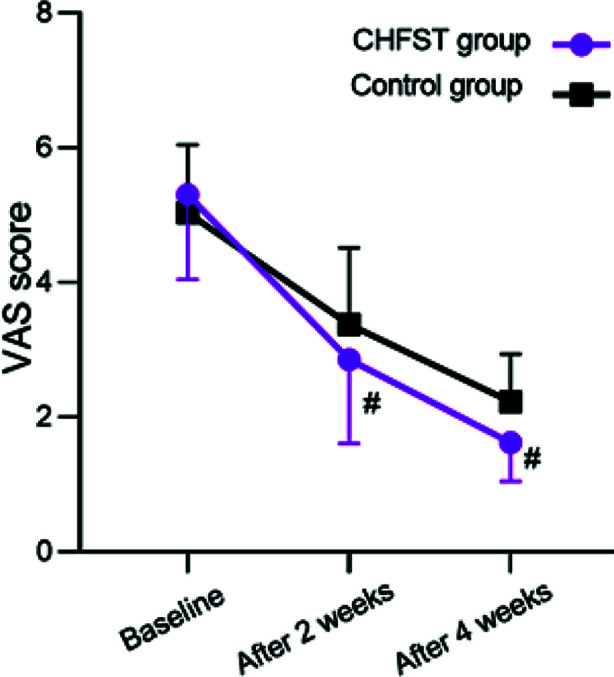
Change curves of VAS scores for two groups; Compared with the control group, #P<0.05.

In this retrospective study, clinical data of 136 patients, including 54 males and 82 females, were analyzed. The age range was 37-81 years old, with an average of 62.3 ± 11.0 years old. Sixty-eight patients who received standard CHFST plus functional exercise (CHFST group) were matched in a 1:1 ratio with a cohort receiving routine treatment plus functional exercise (Fig.1). This was not propensity score matching (PSM); instead, we used direct one-to-one manual matching based on the following clinical variables: gender, age, BMI, comorbidities (hypertension, diabetes, coronary heart disease), smoking status, alcohol consumption, stroke type, affected side and stroke duration. Baseline characteristics are shown in Table-I. No statistically significant differences were found between the two groups in any of these variables, indicating comparability. There was no statistically significant difference in baseline VAS scores between the two groups (5 (4.5-6) vs. 5 (5-6)) (P>0.05).; After two and four weeks of intervention, the VAS scores of the two groups were significantly reduced compared to baseline and considerably lower in the CHFST group than the control group (P<0.05) (Fig.2).

**Table-I:** Comparison of basic clinical data.

Index	CHFST group (n=68)	Control group (n=68)	χ^2^/t/Z	P
Female (yes), n(%)	39 (57.4)	43 (63.2)	0.491	0.483
Age (year), mean±SD	62.3±10.0	63.5±11.9	-0.663	0.509
BMI (kg/m²), mean±SD	23.0±2.6	23.7±3.4	-1.300	0.196
Hypertension (yes), n(%)	45 (66.2)	48 (70.6)	0.306	0.580
Diabetes (yes), n(%)	35 (51.5)	28 (41.2)	1.449	0.229
Coronary heart disease (yes), n(%)	30 (44.1)	35 (51.5)	0.737	0.391
Smoking (yes), n(%)	25 (36.8)	33 (48.5)	1.924	0.165
Drinking alcohol (yes), n(%)	27 (39.7)	26 (38.2)	0.031	0.860
Types of stroke, n(%)			1.093	0.296
Cerebral infarction	43 (63.2)	37 (54.4)		
Cerebral hemorrhage	25 (36.8)	31 (45.6)		
Affected side, n(%)			0.121	0.727
Left	41 (60.3)	39 (57.4)		
Right	27 (39.7)	29 (42.6)		
Stroke course (months)	52.5 (42.5, 62.5)	55.5 (46, 68.5)	-1.374	0.169

There was no statistically significant difference in the pretreatment levels of shoulder flexion, shoulder extension, shoulder abduction, shoulder external rotation range of motion, FMA-UE and WHOQOL-BREF scores between the two groups (P>0.05). After four weeks of treatment, both groups showed significant improvements in shoulder flexion (116 (102.5-128.5) vs. 99.5 (95-107)), shoulder extension (47 (43-53) vs. 44 (37-50)), shoulder abduction (106.5 (96.5-121.5) vs. 101.5 (92.5-113)), shoulder external rotation range of motion (60.5 (49-75) vs. 54 (46-68.5)), FMA-UE (38.5 (34.5-43.5) vs. 34 (30-39)) and WHOQOL-BREF scores (96.6 ± 14.5 vs. 91.2 ± 9.8) compared to baseline (P<0.05). Patients in the CHFST group showed more significant improvements compared to the control group (P<0.05) (Table-II).

**Table-II:** Comparison of shoulder joint range of motion, FMA-UE and WHOQOL-BREF scores.

Time	CHFST group (n=68)	Control group (n=68)	Z/t	P
***Baseline***				
Shoulder flexion	74 (66-89.5)	74 (65-84)	-0.668	0.504
Shoulder extension	35.5 (31-42)	35 (27.5-41.5)	-0.868	0.385
Shoulder abduction joint	88 (83-96)	94.5 (85-102)	-1.659	0.097
Shoulder external rotation range of motion	47.5 (38-60.5)	45 (38-58.5)	-0.952	0.341
FMA-UE score	21 (18-25.5)	20 (16.5-25)	-1.525	0.127
WHOQOL-BREF score	78.6±10.2	81.2±11.3	-1.453	0.148
***After four weeks of treatment***				
Shoulder flexion	116 (102.5-128.5)	99.5 (95-107)	-4.119	<0.001
Shoulder extension	47 (43-53)	44 (37-50)	-2.994	0.003
Shoulder abduction joint	106.5 (96.5-121.5)	101.5 (92.5-113)	-2.151	0.032
Shoulder external rotation range of motion	60.5 (49-75)	54 (46-68.5)	-2.271	0.023
FMA-UE score	38.5 (34.5-43.5)	34 (30-39)	-4.289	<0.001
WHOQOL-BREF score	96.6±14.5	91.2±9.8	2.536	<0.001

## DISCUSSION

This study showed that the combination of CHFST and functional exercise is more efficient in reducing the VAS score, improving shoulder joint mobility and upper limb function and enhancing QOL of HSP patients compared to the conventional treatment.

Beyond statistical significance, the observed improvements in VAS and FMA-UE also exceeded the minimal clinically important differences (MCIDs) reported in the literature. Previous studies have suggested that a reduction of 1.0-1.5 points in VAS, or ≥1.4 points in stroke rehabilitation, reflects a clinically meaningful improvement in pain perception.^18^,^19^ In our study, patients in the CHFST group achieved a reduction of approximately 2-3 points, which clearly surpasses this threshold. Similarly, the MCID for the upper-limb Fugl-Meyer Assessment (FMA-UE) has been reported to range from 4 to 7 points depending on recovery stage.^20^,^21^ The mean improvement of about 17-18 points in our CHFST group far exceeded this benchmark, supporting the interpretation that CHFST combined with exercise yields clinically meaningful functional gains.

These results confirm previous research. Guo et al.^22^ demonstrated promising prospects of CHFST in the treatment of neuropathic pain. Fu et al.^23^ found that CHFST can alleviate swelling and pain after fracture surgery and reduce the occurrence of complications. Chen et al.^24^ showed that the combination of CHFST and exercise rehabilitation training can effectively alleviate the pain level of patients with ankle fractures after surgery, reduce ankle swelling, improve the contraction force and muscle coordination of the ankle dorsiflexor and plantar flexor muscle groups.

At present, there are many treatment methods for HSP after stroke, including anti-inflammatory, analgesic and nerve nourishing of western medicine, as well as acupuncture and moxibustion, massage, rehabilitation training and physical therapy of TCM, with varying efficacy.^6^,^15^-^17^ Recent international evidence, including the European Stroke Organisation (ESO) consensus framework on motor rehabilitation (2023) and systematic reviews and meta-analyses on hemiplegic shoulder pain, also highlight the ongoing lack of consensus regarding optimal interventions, particularly in chronic cases.^25^-^27^ These reports emphasize the need for individualized, multimodal approaches that integrate physical, pharmacological, and complementary therapies. A study by Lees et al.^28^ found that the functional recovery of stroke patients is related to the timing of early rehabilitation treatment initiation.^28^,^29^ Previous studies emphasized that proper limb placement, correct transfer and appropriate suspension are the foundation of rehabilitation training for HSP that not only fixes the relaxed shoulder joint in the correct position, but also reduces the stimulation of the shoulder joint on the limbs, relieving pain.^14^,^15^,^24^ Rehabilitation exercise of the affected limb, in addition to treating the root cause of the disease, can effectively suppress abnormalities in the shoulder joint muscle group, reduce shoulder strap muscle spasms and effectively alleviate shoulder severity.^15^,^24^ Furthermore, rehabilitation training combined with other treatments (such as acupuncture, moxibustion and electrical stimulation) can effectively improve the pain symptoms and limb dysfunction of HSP patients.^30^,^31^

In TCM theory, pain is believed to be caused by qi stagnation, blood stasis and occlusion of the meridians.^17^,^22^-^24^ Post stroke, there is stasis in the meridians, loss of muscle nourishment and loss of bone joint recording, leading to pain.^22^,^24^ Therefore, according to the principles of TCM treatment, effective HSP treatment necessitates relaxing muscles and activating collaterals, promoting blood circulation and removing stasis and relieving pain and congestion.^32^,^33^ The alkaloids and flavonoids contained in herbs such as Chuanxiong Rhizome (Ligustici Chuanxiong Rhizoma), Safflower (Carthami Flos), Weilingxian (Clematidis Radix et Rhizoma) and Mugwort Leaf (Artemisiae Argyi Folium), used in this study, can dilate blood vessels, increase shoulder tissue blood flow and promote the clearance of inflammatory metabolites.^32^-^36^

The volatile oil components of Duhuo (Angelicae Pubescentis Radix), Papaya Fruit (Chaenomelis Fructus), Qianghuo (Notopterygii Rhizoma et Radix), Cinnamon Twig (Cinnamomi Ramulus), Mulberry Twig (Mori Ramulus), etc. can inhibit prostaglandin synthesis and reduce the sensitivity of pain receptors;^33^-^36^ Frankincense (Olibanum), Asarum (Asari Radix et Rhizoma), etc. can relieve pain; Medications such as Stretching Muscle Grass and Bone Penetrating Grass can enhance collagenase activity, improve the elasticity of tendons and joint capsules and restore joint mobility. Cinnamomum cassia and Asarum can open the drug permeation channels in the skin.^33^-^38^ Ginger (Zingiber officinale), Rice Wine and salt can be used as auxiliary materials or medicinal additives to achieve the goals of “increasing efficiency, reducing toxicity and promoting menstruation”.^36^-^38^

CHFST is a traditional external treatment method in TCM. Through the thermal effect of water vapor, drugs can be directly applied to the affected area, improving local blood circulation. The effective drug ingredients enter the body through the skin and muscles, promoting metabolism and improving local tissue nutrition, thereby relieving pain and improving shoulder joint mobility.^36^-^40^ The results of this study also showed that after the combination of CHFST and functional exercise, the VAS scores of the CHFST group were significantly lower than those of the control group after two and four weeks of treatment, while the shoulder joint mobility was considerably higher. This is consistent with the findings of Tan et al.^41^ and Cui et al.^42^

To compare our findings within an international therapeutic context, it is necessary to compare CHFST with widely accepted approaches for hemiplegic shoulder pain. In recent years, botulinum toxin injection has been regarded as an effective intervention to reduce post-stroke spasticity and shoulder pain, although its benefits are often temporary, requiring repeated administrations and carrying the risks of transient muscle weakness and injection-site discomfort.^43^ Electrical stimulation, including NMES, TENS, and FES, has also been shown to provide short-term analgesia and promote motor relearning; however, its long-term effect is frequently attenuated by neuromuscular habituation, and there is marked variability in stimulation protocols across trials.^44^ Suprascapular nerve block represents another option that can offer rapid pain relief and facilitate early rehabilitation participation, but the procedure may lead to complications such as bleeding, local numbness, or weakness of the rotator cuff, and repeated interventions are often required to sustain analgesia.^45^ Against this backdrop, CHFST presents itself as a non-invasive, relatively low-cost, and well-tolerated modality that may be particularly attractive for patients who are unwilling or unable to undergo injections or electrical stimulation. Nonetheless, unlike the aforementioned therapies that have been tested in multiple randomized or multicenter trials, CHFST currently lacks robust head-to-head comparisons with international standards. While our results suggest potential advantages of combining CHFST with functional exercise, its definitive role in global rehabilitation practice should be determined by future multicenter prospective studies with rigorous outcome measures, including pain, function, quality of life, and safety.

In this study, after four weeks of treatment, CHFST combined with functional exercise resulted in significantly higher FMA-UE and WHOQOL-BREF scores compared to the traditional treatment approach. It is plausible that the warm stimulation of 40-45°C during fumigation dilates blood vessels around the shoulder, increases blood flow velocity, accelerates the excretion of metabolic waste and promotes the transport of nutrients to damaged tissues.^40^,^42^ Furthermore, thermal stimulation may reduce patients’ pain sensitivity during functional exercises. Therefore, performing functional exercises within 30 minutes after fumigation further enhances the muscle relaxation effect of thermal stimulation, allows for greater joint mobility, prevents disuse atrophy caused by pain-avoidant movements and improves the recovery of limb motor function.^33^-^38^ Additionally, more effective pain relief will ultimately increase the range of motion of the shoulder joint and improve the upper limb motor function, as well as the overall QOL of HSP patients.

Based on the inclusion criteria, most participants in this study presented with mild to moderate hemiplegic shoulder pain (HSP) and were capable of completing regular functional exercise. Therefore, the therapeutic effects observed are most applicable to this subgroup. Notably, Chinese herbal fumigation steam therapy (CHFST) may be particularly beneficial for patients who are unable or unwilling to undergo acupuncture, electrical stimulation, nerve blocks, or repeated corticosteroid injections. As a non-invasive and generally well-tolerated intervention, CHFST offers a promising adjunct or alternative for individuals who cannot tolerate invasive treatments. Future research should evaluate the efficacy of CHFST across broader HSP populations, including patients with severe pain, complex comorbidities, or limited physical tolerance.

Additionally, although this study demonstrated statistically significant improvements in the total WHOQOL-BREF score, domain-level analysis (i.e., physical, psychological, social, and environmental domains) could not be conducted due to limitations in the retrospective dataset. Specifically, only the total score was consistently available in clinical records, without individual domain data. We acknowledge this as a methodological limitation. Prospective studies should be designed to systematically collect and analyze subdomain-specific data from the WHOQOL-BREF, which will allow for a more detailed understanding of the therapy’s impact on different dimensions of quality of life.

### Strength of this study.

First, it is among the first to systematically evaluate the combined use of Chinese herbal fumigation steam therapy (CHFST) and functional exercise in patients with post-stroke hemiplegic shoulder pain (HSP). Second, multiple clinically relevant outcomes were assessed-including pain severity (VAS), shoulder range of motion (ROM), upper limb motor function (FMA-UE), and quality of life (WHOQOL-BREF)-providing a comprehensive and multidimensional evaluation. Third, the findings suggest that CHFST may serve as a non-invasive, economical, and well-tolerated adjunctive therapy, particularly for patients who are unsuitable for more invasive treatments such as nerve blocks or botulinum toxin injections.

Future studies should build on these findings by conducting multi-center, prospective, large-scale trials with extended follow-up periods (≥3-6 months) to verify the long-term efficacy and recurrence prevention of CHFST. These studies should incorporate systematic adverse event monitoring, dermatologic evaluations, and documentation of treatment adherence, dose modifications, or discontinuations. Investigations should also focus on efficacy across different patient subgroups (e.g., mild vs. severe pain, patient’s intolerant of acupuncture/electrical stimulation), and include head-to-head comparisons with internationally recognized treatment modalities.

In summary, this study confirms the effectiveness of CHFST combined with functional exercise for HSP. This intervention method is safe and easy to implement, especially in patients who are weak and not suitable for acupuncture and moxibustion. The warm effect of CHFST can dilate blood vessels, increase local blood flow and create better tissue conditions for functional exercise.^31^-^35^ At the same time, functional exercise can promote the absorption and distribution of effective ingredients in medication, forming a pharmacological-physical dual intervention mode that is highly conductive for improving shoulder pain.^14^,^15^,^24^,^33^-^38^

### Limitations:

First, it was a single-center retrospective analysis with a relatively small sample size, which limits the generalizability and robustness of the findings. Second, the outcome assessment was limited to a 4-week follow-up period. Given the fluctuating nature of hemiplegic shoulder pain (HSP) during rehabilitation, this short-term observation may not fully reflect the persistence of treatment benefits or the risk of recurrence. Future prospective, multi-center studies with extended follow-up (≥3-6 months) are needed to evaluate long-term efficacy. Third, the pathophysiology of HSP is multifactorial and complex, often involving flaccid paralysis in the early stages of stroke, shoulder subluxation, and soft tissue imbalance. Although CHFST showed promising results in this cohort, the precise mechanisms remain unclear.

Further research is needed to explore its pharmacological pathways, as well as the optimal composition, concentration, and frequency of the treatment protocol. Fourth, due to the retrospective nature of the study, several methodological limitations should be noted. Although groups were matched based on key demographic and clinical characteristics, no formal statistical matching method such as propensity score matching (PSM) was used, and standardized mean differences (SMDs) or Love plots were not calculated. These omissions may affect the assessment of covariate balance and introduce residual confounding. We recommend that future prospective studies incorporate PSM or similar methods, along with explicit diagnostics for covariate balance. Fifth, the choice of statistical methods was constrained by sample size and data structure. Non-parametric tests (e.g., Wilcoxon and Mann-Whitney U tests) were used to accommodate non-normal distributions.

Although sensitivity analyses-including subgroup stratification and outlier exclusion-were performed to verify the robustness of findings, advanced modeling techniques (e.g., mixed-effects models or baseline-adjusted ANCOVA) were not applied. Future studies should use such techniques with full model diagnostics to improve analytical rigor. Sixth, only total WHOQOL-BREF scores were available; domain-level scores were not recorded in clinical documentation. This limited our ability to evaluate CHFST’s differential impact on physical, psychological, social, and environmental domains of quality of life. This should be addressed in future studies through systematic data collection. Seventh, although a small baseline difference in shoulder abduction was observed (P = 0.097), we believe this did not substantially bias post-treatment comparisons.

Nonetheless, baseline-adjusted analyses (e.g., ANCOVA) were not performed due to the limited sample and data constraints, which we recognize as a limitation. Eighth, formal reliability testing (e.g., intraclass correlation coefficients [ICCs]) and assessor blinding were not conducted. Since CHFST was visibly delivered, complete blinding of outcome assessors was not feasible, possibly introducing expectation bias. This methodological limitation has been acknowledged. Finally, adverse event (AE) monitoring was not systematic. While no major skin-related AEs (e.g., burns or allergic reactions) were recorded, minor reactions may have been missed. Dermatologic assessments, dose adjustments, and treatment discontinuation data were not collected. Future prospective studies should incorporate standardized safety monitoring and AE reporting protocols to comprehensively evaluate the tolerability and safety profile of CHFST.

## CONCLUSION

The combination of CHFST and functional exercise is a safe and feasible treatment method that shows potential in alleviating pain, improving shoulder joint mobility and upper limb function, and enhancing the quality of life (QOL) of patients with hemiplegic shoulder pain (HSP). Future multi-center studies with larger sample sizes and longer follow-ups are warranted to confirm these findings, and to further explore the optimal formula composition, treatment frequency, and the potential synergistic effects of CHFST when combined with other modalities such as electrical stimulation and pulsed radiofrequency.

### Authors’ contributions:

**GQ:** Literature search, study design and manuscript writing.

**GQ and LH:** Data collection, data analysis and interpretation. Critical Review.

**GQ:** Manuscript revision and validation and is responsible for the integrity of the study.

All authors have read and approved the final manuscript.
